# Experience with poorly myelosuppressive chemotherapy schedules for advanced myeloma. The Cooperative Group of Study and Treatment of Multiple Myeloma.

**DOI:** 10.1038/bjc.1996.138

**Published:** 1996-03

**Authors:** S. Brugnatelli, A. Riccardi, G. Ucci, O. Mora, L. Barbarano, N. Piva, L. Piccinini, C. Bergonzi, A. De Paoli, M. Di Stasi, E. Rinaldi, G. Trotti, M. Petrini, E. Ascari

**Affiliations:** Medicina Interna ed Oncologia Medica, Universita and Istituto di Ricovero e Cura a Carattere Scientifico Policlinico S. Matteo, Pavia, Italy.

## Abstract

In a multicentre study, 83 patients with advanced and previously uniformly treated multiple myeloma (MM) were randomised between cyclophosphamide (600 mg m-2) and epirubicin (70 mg m-2), administered every 3 weeks for three courses and both associated with prednisone and interferon-alpha2b. Both regimens were administered on an outpatient basis and had low haematological toxicity. Clinical results were similar. Overall response rate (43%) and median response and survival (5.9 and 14.1 months respectively) compare well with those obtained with more aggressive chemotherapy schedules.


British Journal of Cancer (1996) 73, 794-797
© 1996 Stockton Press All rights reserved 0007-0920/96 $12.00

### Experience with poorly myelosuppressive chemotherapy schedules for advanced myeloma

S Brugnatelli[1], A Riccardi[1], G Ucci[1], O Mora[1], L Barbarano[2], N Piva[3], L Piccinini[4], C Bergonzi[5], A De Paoli[6], M Di Stasi[7], E Rinaldi[8], G Trotti[9], M Petrini[10] and E Ascari[1] (for the Cooperative Group of Study and Treatment of Multiple Myeloma)

*[1]Medicina Interna ed Oncologia Medica, Università and Istituto di Ricovero e Cura a Carattere Scientifico Policlinico S. Matteo, 27100 Pavia; [2]Divisione di Ematologia, Ospedale di Niguarda, 20100 Milano; [3]Istituto di Ematologia, Università di Ferrara, 44100 Ferrara; [4]Istituto di Oncologia, Università di Modena, 41100 Modena; [5]Divisione di Medicina II, Ospedale di Cremona, 26100 Cremona; [6]Divisione di Medicina II, Ospedale di Legnano, 20025 Legnano; [7]Divisione di Medicina I, Ospedale di Piacenza, 29100 Piacenza; [8]Divisione di Medicina I, Ospedale di Magenta, 20013 Magenta; [9]Medicina Generale II, Ospedale di Busto Arsizio, 21052 Busto Arsizio; [10]Istituto di Ematologia, Università di Pisa, 56100 Pisa, Italy.*

**Summary** In a multicentre study, 83 patients with advanced and previously uniformly treated multiple myeloma (MM) were randomised between cyclophosphamide (600 mg $m^{-2}$) and epirubicin (70 mg $m^{-2}$), administered every 3 weeks for three courses and both associated with prednisone and interferon-α2b. Both regimens were administered on an outpatient basis and had low haematological toxicity. Clinical results were similar. Overall response rate (43%) and median response and survival (5.9 and 14.1 months respectively) compare well with those obtained with more aggressive chemotherapy schedules.

**Keywords:** advanced myeloma; outpatient therapy; randomisation; epirubicin; cyclophosphamide

Treatment of advanced multiple myeloma (MM) usually employs combination chemotherapy (Buzaid and Durie, 1988). We used either cyclophosphamide (CTX) or epirubicin (EPI), both associated with recombinant interferon (IFN) and prednisone (P), as third-line therapy, with the expectancy that haematological toxicity would be low and the therapy feasible on an outpatient basis. All patients came from Protocol MM87 (Riccardi *et al.*, 1994), where they were treated, as first-line therapy, either with melphalan and prednisone (MPH-P) or peptichemio (PTC), vincristine (VCR) and P. As second-line therapy, patients resistant to or relapsed following one combination were crossed to the other combination.

The choice of salvage CTX came from the fact that MPH-resistant MM patients may respond to this drug (Bergsagel *et al.*, 1972; Lenhard *et al.*, 1984). The use of EPI was justified by the response of advanced patients to the several combination chemotherapies including anthracycline (Alberts *et al.*, 1976; Finnish Leukaemia Group, 1990).

### Materials and methods

Between January 1989 and December 1993, 83 consecutive patients (Table I) entered a third-line, prospective, multicentre, randomised protocol (Protocol MM87/01) for advanced MM. Patients were primarily resistant to or relapsed following a response to first- and second-line therapies of Protocol MM87 (i.e. to MPH-P and PTC-VCR-P) (Riccardi *et al.*, 1994).

Randomisation was between EPI (70 mg $m^{-2}$) and CTX (600 mg $m^{-2}$) given by i.v. infusion on day 1 every 3 weeks for 3 courses. Both cytostatics were combined with P (2 mg $kg^{-1}$ $day^{-1}$, days 1-4 and 11-15) and IFN-α2b (3 MU three times a week).

Correspondence: A Riccardi, Cattedra di Oncologia Medica, Medicina Interna ed Oncologia Medica, Policlinico S. Matteo, 27100 Pavia, Italy
Received 7 June 1995; revised 5 October 1995; accepted 31 October 1995

### *Response, maintenance therapy and relapse*

Response was evaluated at the end of induction therapy, according to slightly modified clinical criteria (Riccardi *et al.*, 1994) adopted by the SECSG (Cohen *et al.*, 1979).

Responsive patients continued therapy until maximum reduction in monoclonal component (MC) (i.e. the plateau phase) was reached and maintained for 6 months, with stable clinical, haematological and radiological conditions. Then, they continued only on IFN-α2b (3 MU three times a week) as a maintenance therapy.

Relapse was defined as a >50% increase in the plateau level of MC and/or an increase in the size and/or number of skeletal lytic lesions.

### *Follow-up and statistical evaluation*

The guidelines for following up MM are similar to those detailed elsewhere (Riccardi *et al.*, 1994). To define the drug toxicity blood counts were performed twice in the interval between courses.

The statistical evaluation of the differences in response rate and duration of response (from the end of successful induction therapy until relapse) and of survival (from randomisation to death) are described elsewhere (Riccardi *et al.*, 1994).

### Results

In both EPI-P-IFN and CTX-P-IFN arms, patients were similar for the main clinical characteristics (Table I), and more of them had received MPH-P as a first-line therapy, with similar response rate.

Patients who relapsed following a response to first-line therapy had received a median of 19.8 (range 12-33) and of 17.1 (10-28) courses of MPH-P and PTC-VCR-P respectively. In patients who were primarily resistant, the corresponding figures were 12.1 (10-16) and 12.4 (8-16).

### *Response*

Response was evaluated in 70/83 (85%) patients (Table II), including four patients (two from each arm) who died before

**Table I** Main clinical characteristics of patients with advanced multiple myeloma who were randomised to be treated, as third-line therapy, with the combination of epirubicin, prednisone and recombinant interferon-α2b (EPI-P-IFN) or with the combination of cyclophosphamide, prednisone and recombinant interferon-α2b (CTX-P-IFN)

| | *EPI-P-IFN* | *CTX-P-IFN* | *Overall* |
|---|---|---|---|
| Number of patients | 43 | 40 | 83 |
| Male/Female | 21/22 | 20/20 | 41/42 |
| Median age (years) (range) | 58 (46-75) | 62 (44-79) | 61 (44-79) |
| IgG | 35 | 25 | 60 |
| IgA | 7 | 12 | 19 |
| LC | 1 | 3 | 4 |
| K | 26 | 25 | 51 |
| L | 17 | 15 | 32 |
| $\beta$-2 $\mu g\,ml^{-1}$, median (range) | 5.1 (1.4-13.7) | 4.8 (2.1-11.3) | 4.9 (1.4-13.7) |
| Lytic lesions | | | |
| 0-3 (%) | 6 (14) | 9 (22) | 15 (18) |
| >3 (%) | 8 (19) | 5 (13) | 13 (16) |
| with pathological fractures (%) | 29 (67) | 26 (65) | 55 (66) |
| Hb, $g\,dl^{-1}$ | | | |
| >9 | 30 | 26 | 56 |
| ≤9 | 13 | 14 | 27 |
| Serum creatinine, $mg\,dl^{-1}$ | | | |
| ≤2 | 41 | 39 | 80 |
| >2 | 2 | 1 | 3 |
| Prior first-line therapy | | | |
| MPH-P, no. of patients | 30 | 33 | 63 |
| PR+CR (%) | 34 | 40 | 37 |
| NR (%) | 66 | 60 | 63 |
| PTC-VCR-P, no. of patients | 13 | 7 | 20 |
| PR+CR (%) | 54 | 43 | 50 |
| NR (%) | 46 | 57 | 50 |

MPH-P, melphalan-prednisone; PTC-VCR-P, peptichemio-vincristine-prednisone; PR, partial response; CR, complete response; NR, no response (stable+progressive disease).

**Table II** Response of patients with advanced multiple myeloma to the combination of epirubicin, prednisone and recombinant interferon-α2b (EPI-P-IFN) or to the combination of cyclophosphamide, prednisone and recombinant interferon-α2b (CTX-P-IFN)

| | *EPI-P-IFN* | *CTX-P-IFN* | *Overall* |
|---|---|---|---|
| Evaluable patients | 37 | 33 | 70 |
| Relapsed patients[a] | 14 | 13 | 27 |
| Resistant patients[b] | 23 | 20 | 43 |
| CR+PR (%) | 14/37 (38) | 16/33 (48) | 30/70 (43) |
| In relapsed patients (%)[a] | 4/14 (28) | 6/13 (46) | 10/27 (37) |
| In resistant patients (%)[b] | 10/23 (43) | 10/20 (50) | 20/43 (47) |
| PR (%) | 8/37 (22) | 13/33 (40) | 21/70 (30) |
| CR (%) | 6/37 (16) | 3/33 (8) | 9/70 (12) |
| SD (%) | 16/37 (43) | 13/33 (40) | 29/70 (42) |
| PD (%) | 7/37 (19) | 4/33 (12) | 11/70 (16) |

[a]Relapsed patients are those patients who relapsed following a response to first-line therapy with MPH-P or with PTC-VCR-P. [b]Resistant patients are those patients who were primarily resistant to both MPH-P and PTC-VCR-P as first- and second-line therapies. PD, progressive disease (other abbreviations as in Table I)

response could be established and were considered as non-responders. Thirteen patients were not evaluated for refusal to continue treatment (four patients), insufficient data or lost to follow-up (nine patients).

The overall response rate was 43%, without statistical difference between the EPI-P-IFN (38%) and the CTX-P-IFN (48%) arm.

The response rate was similar in patients firstly treated with MPH-P and with PTC-VCR-P.

More responsive patients had WHO/ECOG performance status ameliorated (Table III), in a median time of 7 (range: 6-10) weeks in the EPI-P-IFN and of 10 (range: 6-12) weeks in the CTX-P-IFN arm.

*Duration of response and of survival*

The overall median duration of response was 5.9 months. It was similar in the EPI-P-IFN (5.5 months) and in the CTX-P-IFN (6.4 months) arm.

**Table III** Changes in WHO/ECOG performance status in responder patients with advanced multiple myeloma treated with third-line therapy (EPI-P-IFN or CTX-P-IFN)

| *WHO/ECOG performance status* | *No. of patients* Before therapy | After therapy |
|---|---|---|
| EPI-P-IFN arm (A) | | |
| 0-1 | 4 | 11 |
| 2 | 4 | 3 |
| 3 | 6 | 1 |
| CTX-P-IFN arm (B) | | |
| 0-1 | 6 | 7 |
| 2 | 5 | 6 |
| 3 | 5 | 3 |
| Arm A+Arm B | | |
| 0-1 | 10 | 18 |
| 2 | 9 | 9 |
| 3 | 11 | 4 |

(abbreviations as in Table I)

**Figure 1** Duration of survival in MM patients who were randomised to be treated for third-line therapy with the combination of epirubicin, prednisone and interferon-α2b (EPI–P–IFN) (——) (33 patients, 13 censored) or with the combination of cyclophosphamide, prednisone and interferon-α2b (CTX–P–IFN) (- - - -) (30 patients, 17 censored). *P*-value, not significant.

Overall median survival was 14.1 months. It was similar in the EPI–P–IFN (13.9 months) and in the CTX–P–IFN (14.3 months) arm (Figure 1), as well as in patients who were primarily resistant to first-line therapy (15.0 months) and in those who relapsed following a response (13.4 months).

*Toxicity*

Overall haematological toxicity was low and 88% of courses were administered on an outpatient basis.

Febrile neutropenia occurred in 12% and grade III anaemia and thrombocytopenia in 7% and 5% of patients. These figures were somewhat but not significantly greater in the EPI–P–IFN than in the CTX–P–IFN arm (15%, 10% and 7% *vs* 6%, 4% and 2% respectively).

Grade 2–3 alopecia was distinctly more frequent in the EPI–P–IFN than in the CTX–P–IFN (55% *vs* 9%, $P<0.01$). Grade 2 emesis occurred in 20% and 9% (*P*-value not significant) of patients respectively. Stomatitis was unusual.

Four patients in both arms stopped IFN for grade 3 chills and/or fever, uncontrolled by acetaminophen premedications.

There was no cardiac damage attributable to EPI and no gastrointestinal, psychiatric or metabolic damage attributable to steroids.

### Discussion

In this randomised study, patients with MM who were resistant to or relapsed following MPH–P and PTC–VCR–P achieved similar clinical benefit from being treated with EPI–P–IFN or CTX–P–IFN. In fact, response rate, changes in WHO/ECOG status and response and survival duration were similar with the two regimens.

These results are in keeping with published data on the value of CTX and anthracyclines for advanced MM. Used alone, CTX was effective in a number (Bersagel *et al.*, 1972; Brandes and Israels, 1987), although not in all (Presant and Klahr, 1978; Maclennan and Cuzick, 1985), non-randomised investigations. At present it is incorporated into regimens for refractory disease (Kyle *et al.*, 1975; Steinke *et al.*, 1985). Anthracyclines have not been used as a single agent. However, anthracycline-containing regimens are effective in both relapsed (Alexanian and Deeicer, 1984; Barlogie and Alexanian, 1987; Presant and Klahr, 1978) and primarily resistant (Cornelissen *et al.*, 1994) patients. The clinical role of IFN and steroids in favouring the effectiveness of both EPI and CTX cannot be established in this study.

As expected, haematological toxicity was low, non-haematological toxicity was acceptable and most patients were treated on an outpatient basis.

The overall 14.1 month median survival compares well with the median survivals of 5–22 months (the weighted median is about 10 months) reported in small non-randomised studies on salvage therapy in MM (Bonnet *et al.*, 1984; Lenhard *et al.*, 1984; Steinke *et al.*, 1985; Alexanian *et al.*, 1986; Forgeson *et al.*, 1988; Finnish Leukaemia Group, 1990; Friedenberg *et al.*, 1991; Gimsing *et al.*, 1991; Cornelissen *et al.*, 1994). These usually employed more cytotoxic drug combinations and often required hospitalisation. Median survival is also not better in young patients with advanced disease following autologous bone marrow (BM) or peripheral blood stem cell transplantation (Barlogie *et al.*, 1986; Fermand *et al.*, 1989).

In conclusion, it seems clinically acceptable to treat advanced MM with poorly myelosuppressive regimens based on medium doses of CTX or anthracyclines.

The following centres also participated in this study: Servizio di Oncologia, Ospedale S. Anna, 22100 Como (Dr C Epifani, Dr M Giordano); Divisione di Ematologia, Ospedale di Pesaro, 61100 Pesaro (Dr C Delfini); Medicina I, Ospedale di Alessandria (Dr G Montanaro, Dr A Pagetto); Medicina I, Ospedale di Melegnano (Professor G Santagati, Dr L Dezza); Medicina I, Ospedale di Gallarate (Dr A Ceriani, Dr R Castiglioni); Cattedra di Ematologia, Università di Parma (Professor V Rizzoli, Dr G Dotti); Medicina B, Ospedale di Biella (Professor S Fontana, Dr M Badone); Medicina Generale, Ospedale di Somma Lombardo (Professor M Mainardi, Dr A Daverio); Medicina C, Ospedale di Varese (Dr N Brumana); Medicina A, Ospedale di Varese (Dr G Pinotti).

**Acknowledgements**
Research supported by AIRC (Associazione Italiana per la Ricerca sul Cancro, Milano), by CNR (Consiglio Nazionale dell Ricerche, Roma, Progetto Finalizzato Applicazioni Cliniche della Ricerca Oncologica, grant no. 94. 01195 PF39), by IRCCS (Instituto di Ricovero e Cura a Carattere Scientifico Policlinico San Matteo, Pavia) and by MURST (Ministero dell'Università e della Ricerca Scientifica e Tecnologica, Roma).

### References

ALBERTS DS, DURIE BGM AND SALMON SE. (1976). Doxorubicin/BCNU chemotherapy for multiple myeloma in relapse. *Lancet*, **1**, 926–928.

ALEXANIAN R AND DEEICER R. (1984). Chemotherapy for multiple myeloma. *Cancer*, **53**, 583.

ALEXANIAN R, BARLOGIE B AND DIXON D. (1986). High-dose glucocorticoid treatment of resistant myeloma. *Ann. Intern. Med.*, **105**, 8–11,

BARLOGIE B AND ALEXANIAN R. (1987). Biology and therapy of multiple myeloma. *Acta Haematol.*, **78**, (suppl. 1), 171.

BARLOGIE B, HALL R, ZANDER A, DICKE, K AND ALEXANIAN R. (1986). High-dose melphalan with autologous bone marrow transplantation for multiple myeloma. *Blood*, **67**, 1298–1301.

BERSAGEL DE, COWAN DH AND HASSELBACH R. (1972). Plasma cell myeloma: response of melphalan-resistant patients to high dose intermittent cyclophosphamide. *Can. Med. Assoc. J.*, **107**, 851–855.

BONNET JD, ALEXANIAN R, SALMON SE, HAUT A AND DIXON DO. (1984).Addition of cisplatin and bleomicin to vincristine-carmustine-doxorubicin-prednisone (VBAP) combination in the treatment of relapsing resistant multiple myeloma: a Southwest Oncology Group Study. *Cancer Treat. Rep.*, **68**, 481–485.

BRANDES LJ AND ISRAELS LG. (1987). Weekly low-dose cyclophosphamide and alternate day prednisone: an effective low toxicity regimen for advanced myeloma. *Eur. J. Haematol.*, **39**, 362.

BUZAID AC AND DURIE BGM. (1988). Management of refractory myeloma: a review. *J. Clin. Oncol.*, **6,** 889–905.

COHEN HJ, SILBERMAN HR, LARSEN WE, JOHNSON L, BARTOLUCCI AA AND DURANT JR. (1979). Combination chemotherapy with intermittent 1-3-bis (2-chloroethyl)-1-nitrosourea (BCNU), cyclophosphamide, and prednisone for multiple myeloma. *Blood*, **54,** 824–836.

CORNELISSEN JJ, SONNEVELD P, SCHOESTER M, RAAIJMAKERS HGP, NIEUWENHUIS HK, DEKKER AW AND LOKHRST HM. (1994). MDR-1 expression and response to vincristine, doxorubicin and dexamethasone chemotherapy in multiple myeloma refractory to alkylating agents. *J.Clin. Oncol.*, **12,** 115–119.

FERMAND JP, LEVY Y, GEROTA J, BENBUNAN M, COSSET JM, CASTAIGNE S, SELIGMANN M AND BROUET JC. (1989). Treatment of agressive multiple myeloma by high-dose chemotherapy and total body irradiation followed by blood stem cells autologous graft. *Blood*, **73,** 20–23.

FINNISH LEUKAEMIA GROUP (1990). Intensive chemotherapy with combinations containing anthracyclines for refractory and relapsing multiple myeloma. *Eur. J. Haematol.*, **44,** 120–123.

FORGESON GV, SELBY S, LAKHANI S, ZULIAN G, VINER C, MAITLAND J AND MCELWAIN TJ. (1988). Infused vincristine and adriamycin with high dose methylprednisolone (VAMP) in advanced previously treated multiple myeloma patients. *Br. J. Cancer*, **58,** 469–473.

FRIEDENBERG WR, KYLE RA, KNOSPE WH, BENNETT JM, TSIATIS AA AND OKEN MM. (1991). High-dose dexamethasone for refractory or relapsing multiple myeloma. *Am. J. Hematol.*, **36,** 171–175.

GIMSING P, BJERRUM OW, BRANDT E, ELLEGAARD J, EVENSEN SA, HANSEN MM, HEDENUS M, HIPPE E, KELDSEN N, PALVA I, RODJER S, TALSTAD I, WESTIN J AND WISLOFF F. (1991). Refractory myelomatosis treated with mitoxantrone in combination with vincristine and prednisone (NOP-regiment): a phase II study. *Br. J. Haematol.*, **77,** 73–79.

KYLE RA, SELIGMAN BR, WALLACE HJ, SILVER RT, GLIDEWELL O AND HOLLAND JF. (1975). Multiple myeloma resistant to melphalan (NSC-8806) treated with cyclophosphamide (NSC-26271), prednisone (NSC-10023), and chloroquine (NSC-187208). *Cancer Chemother. Rep.*, **59,** 557–562.

LENHARD RE, OKEN MM, BARNES JM, HUMPHREY RL, JOHN HG AND SILVERSTEIN MN. (1984). High-dose cyclophosphamide: an effective treatment for advanced refractory multiple myeloma. *Cancer*, **53,** 1456–1460.

MACLENNAN ICM AND CUZICK J. (for the Medical Research Council Working Party on Leukemia in Adults). (1985). Objective evaluation of the role of vincristine in induction and maintenance therapy for myelomatosis. *Br. J. Cancer*, **52,** 153–158.

PRESANT CA AND KLAHR C. (1978). Adriamycin, BCNU, cyclophosphamide plus prednisone in melphalan-resistant multiple myeloma. *Cancer*, **42,** 1222.

RICCARDI A, UCCI G, LUONI R, BRUGNATELLI S, MORA O, SPANEDDA R, DE PAOLI A, BARBARANO L, DI STASI M, ALBERIO F, DELFINI C, NICOLETTI G, MORANDI S, RINALDI E, PICCININI L, DE PASQUALE A AND ASCARI E. (1994). Treatment of multiple myeloma according to extension of disease: a prospective, randomized study comparing a less with a more aggressive cytostatic policy. *Br. J. Cancer*, **70,** 1203–1210.

STEINKE B, BUSCH FW, BECHERER C, OSTENDORF P AND WALLER HD. (1985). Melphalan-resistant multiple myeloma: results of treatment according to the M-2 protocol. *Cancer Chemother. Pharmacol.*, **14,** 279–281.


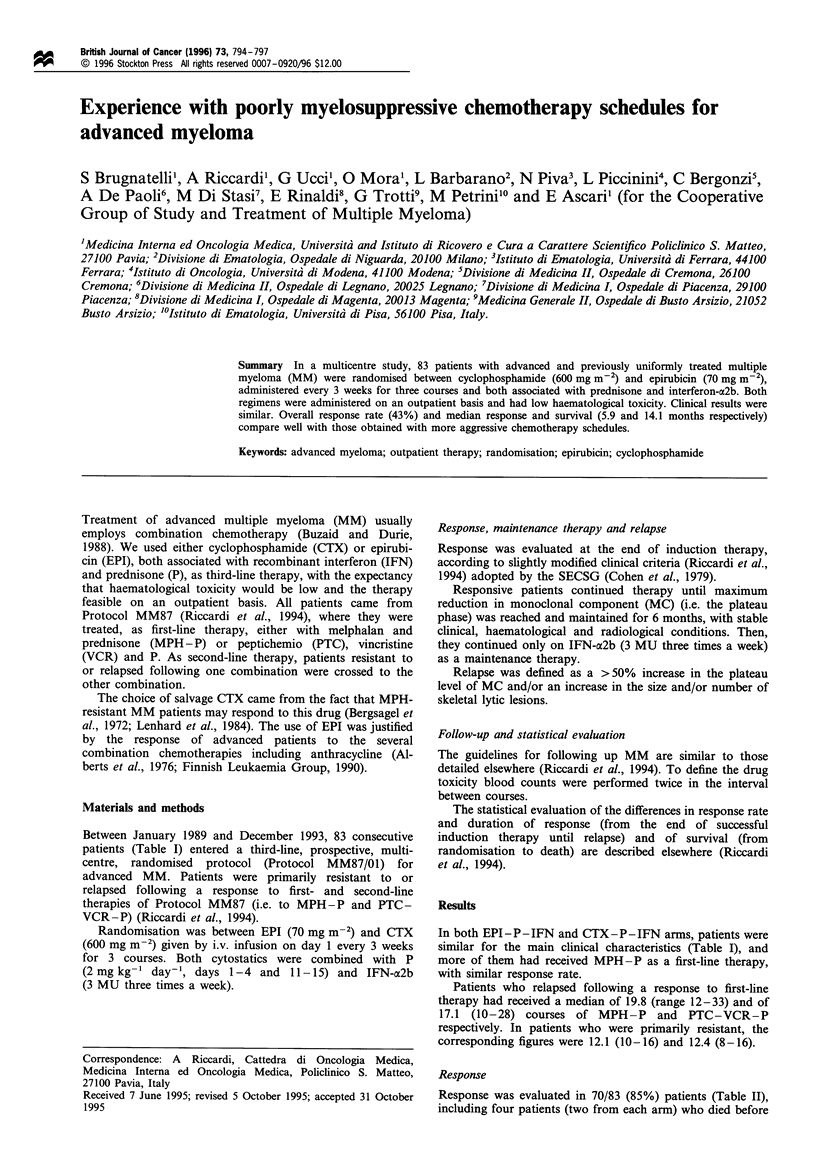

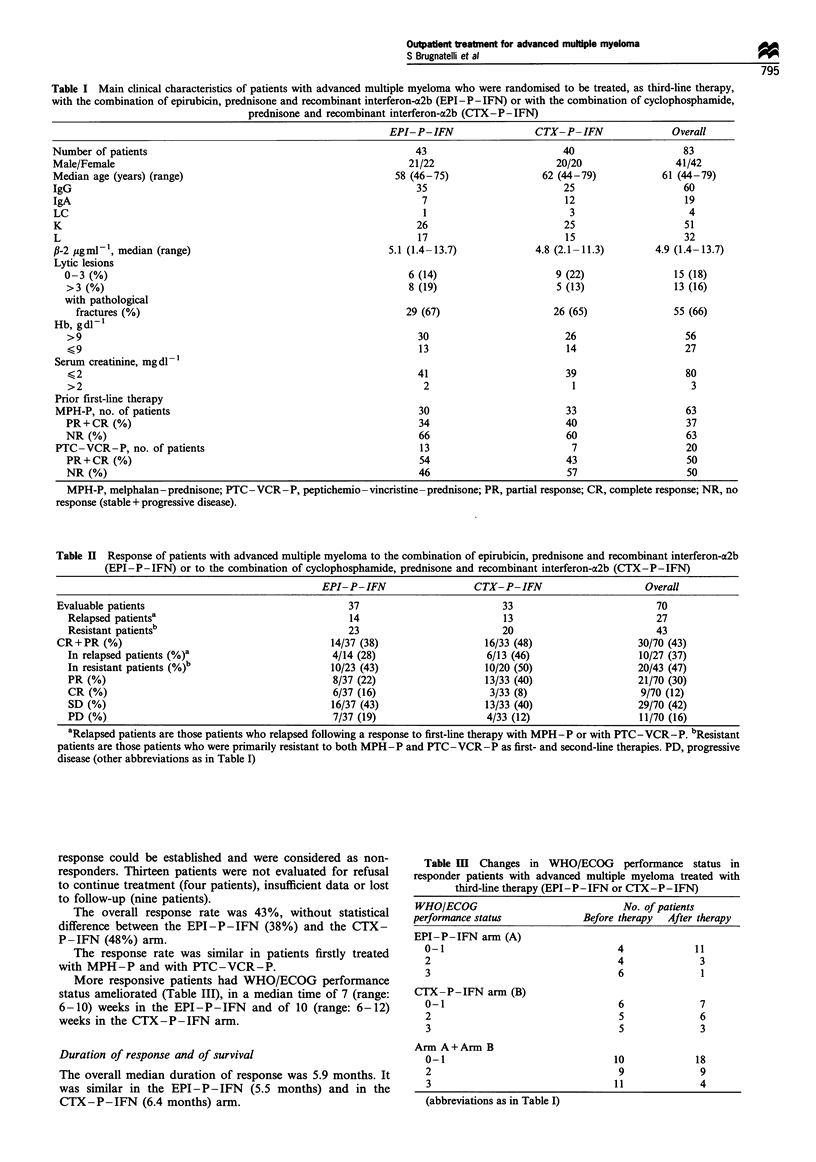

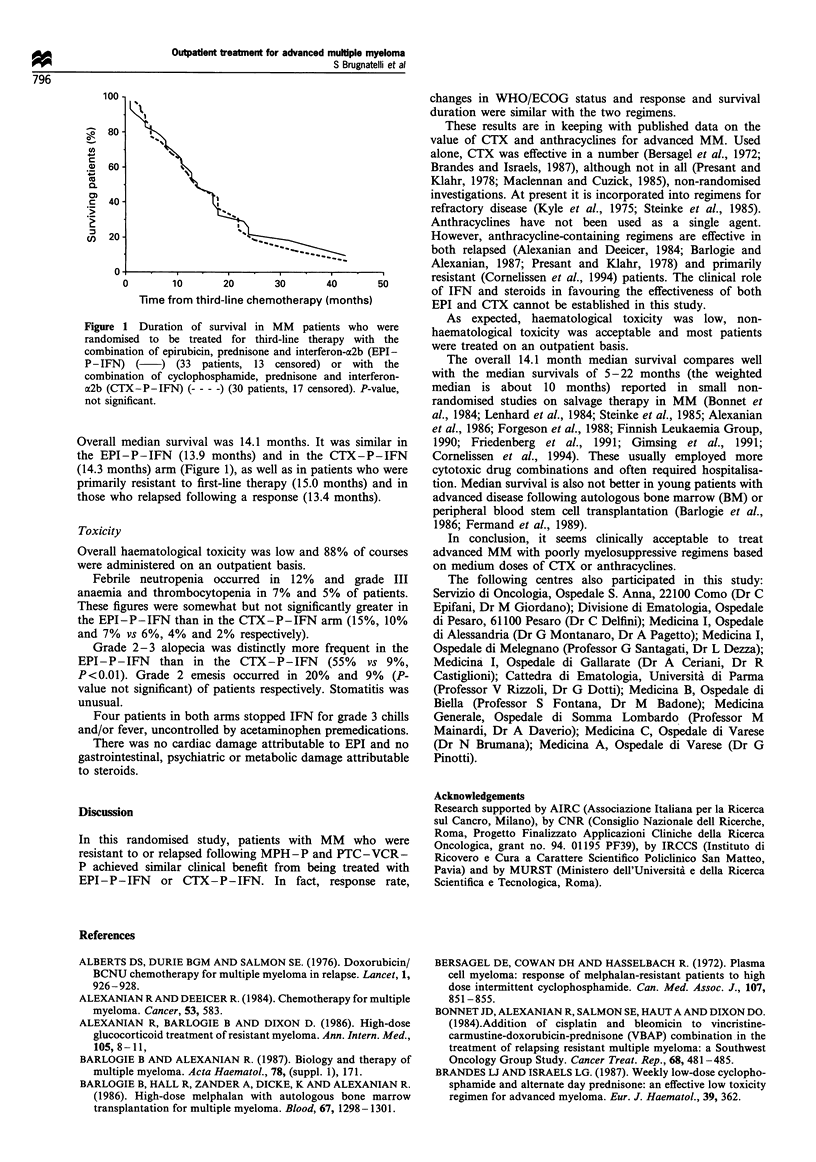

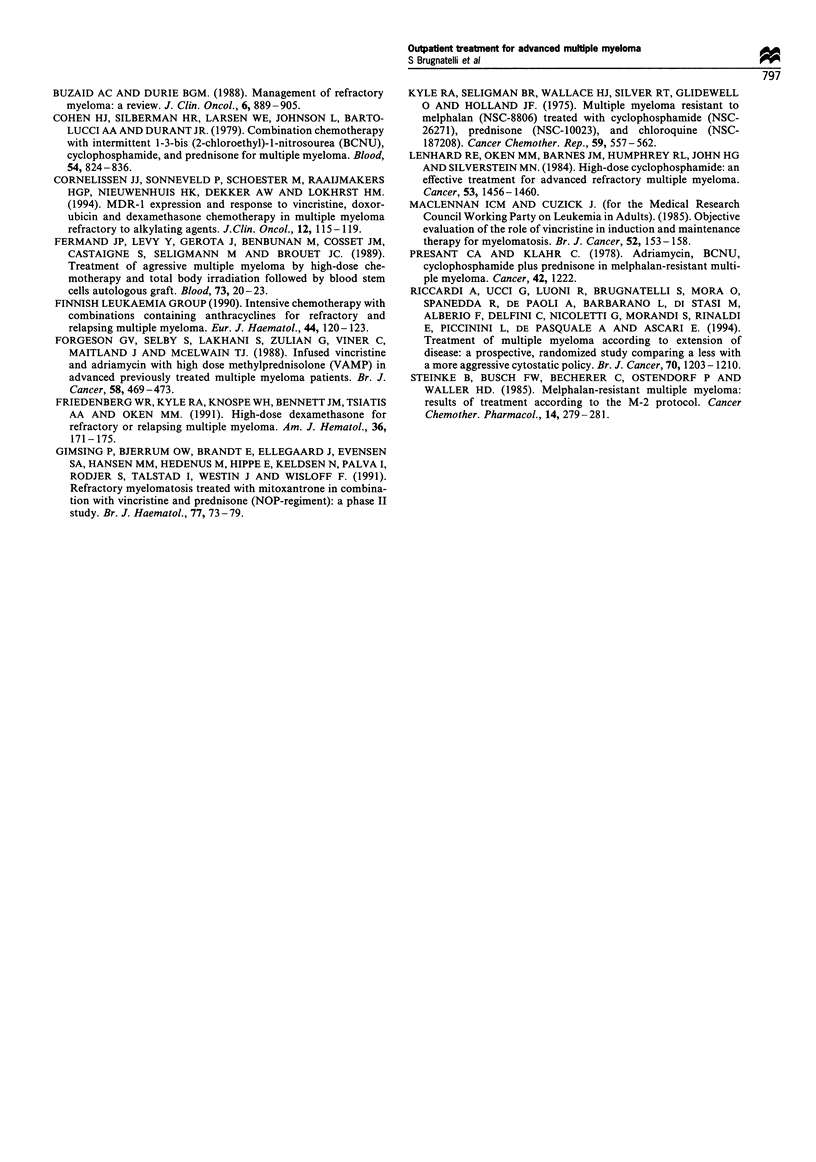


## References

[OCR_00384] Alberts D. S., Durie B. G., Salmon S. E. (1976). Doxorubicin/B.C.N.U. chemotherapy for multiple myeloma in relapse.. Lancet.

[OCR_00393] Alexanian R., Barlogie B., Dixon D. (1986). High-dose glucocorticoid treatment of resistant myeloma.. Ann Intern Med.

[OCR_00391] Alexanian R., Dreicer R. (1984). Chemotherapy for multiple myeloma.. Cancer.

[OCR_00400] Barlogie B., Alexanian R. (1987). Biology and therapy of multiple myeloma.. Acta Haematol.

[OCR_00404] Barlogie B., Hall R., Zander A., Dicke K., Alexanian R. (1986). High-dose melphalan with autologous bone marrow transplantation for multiple myeloma.. Blood.

[OCR_00409] Bergsagel D. E., Cowan D. H., Hasselback R. (1972). Plasma cell myeloma: response of melphalan-resistant patients to high-dose intermittent cyclophosphamide.. Can Med Assoc J.

[OCR_00413] Bonnet J. D., Alexanian R., Salmon S. E., Haut A., Dixon D. O. (1984). Addition of cisplatin and bleomycin to vincristine-carmustine-doxorubicin-prednisone (VBAP) combination in the treatment of relapsing or resistant multiple myeloma: a Southwest Oncology Group study.. Cancer Treat Rep.

[OCR_00422] Brandes L. J., Israels L. G. (1987). Weekly low-dose cyclophosphamide and alternate-day prednisone: an effective low toxicity regimen for advanced myeloma.. Eur J Haematol.

[OCR_00433] Buzaid A. C., Durie B. G. (1988). Management of refractory myeloma: a review.. J Clin Oncol.

[OCR_00435] Cohen H. J., Silberman H. R., Larsen W. E., Johnson L., Bartolucci A. A., Durant J. R. (1979). Combination chemotherapy with intermittent 1-3-bis(2-chloroethyl)1-nitrosourea (BCNU), cyclophosphamide, and prednisone for multiple myeloma.. Blood.

[OCR_00442] Cornelissen J. J., Sonneveld P., Schoester M., Raaijmakers H. G., Nieuwenhuis H. K., Dekker A. W., Lokhorst H. M. (1994). MDR-1 expression and response to vincristine, doxorubicin, and dexamethasone chemotherapy in multiple myeloma refractory to alkylating agents.. J Clin Oncol.

[OCR_00452] Fermand J. P., Levy Y., Gerota J., Benbunan M., Cosset J. M., Castaigne S., Seligmann M., Brouet J. C. (1989). Treatment of aggressive multiple myeloma by high-dose chemotherapy and total body irradiation followed by blood stem cells autologous graft.. Blood.

[OCR_00464] Forgeson G. V., Selby P., Lakhani S., Zulian G., Viner C., Maitland J., McElwain T. J. (1988). Infused vincristine and adriamycin with high dose methylprednisolone (VAMP) in advanced previously treated multiple myeloma patients.. Br J Cancer.

[OCR_00468] Friedenberg W. R., Kyle R. A., Knospe W. H., Bennett J. M., Tsiatis A. A., Oken M. M. (1991). High-dose dexamethasone for refractory or relapsing multiple myeloma.. Am J Hematol.

[OCR_00477] Gimsing P., Bjerrum O. W., Brandt E., Ellegaard J., Evensen S. A., Hansen M. M., Hedenus M., Hippe E., Keldsen N., Palva I. (1991). Refractory myelomatosis treated with mitoxantrone in combination with vincristine and prednisone (NOP-regimen): a phase II study. The Nordic Myeloma Study Group (NMSG). Br J Haematol.

[OCR_00485] Kyle R. A., Seligman B. R., Wallace H. J., Silver R. T., Glidewell O., Holland J. F. (1975). Mutiple myeloma resistant to melphalan (NSC-8806) treated with cyclophosphamide (NSC-26271), prednisone (NSC-10023), and chloroquine (NSC-187208).. Cancer Chemother Rep.

[OCR_00492] Lenhard R. E., Oken M. M., Barnes J. M., Humphrey R. L., Glick J. H., Silverstein M. N. (1984). High-dose cyclophosphamide. An effective treatment for advanced refractory multiple myeloma.. Cancer.

[OCR_00497] MacLennan I. C., Cusick J. (1985). Objective evaluation of the role of vincristine in induction and maintenance therapy for myelomatosis. Medical Research Council Working Party on Leukaemia in Adults.. Br J Cancer.

[OCR_00503] Presant C. A., Klahr C. (1978). Adriamycin, 1,3-bis (2-chloroethyl) 1 nitrosourea (BCNU, NSC No. 409962), cyclophosphamide plus prednisone (ABC-P) in melphalanresistant multiple myeloma.. Cancer.

[OCR_00506] Riccardi A., Ucci G., Luoni R., Brugnatelli S., Mora O., Spanedda R., De Paoli A., Barbarano L., Di Stasi M., Alberio F. (1994). Treatment of multiple myeloma according to the extension of the disease: a prospective, randomised study comparing a less with a more aggressive cystostatic policy. Cooperative Group of Study and Treatment of Multiple Myeloma.. Br J Cancer.

[OCR_00514] Steinke B., Busch F. W., Becherer C., Ostendorf P., Waller H. D. (1985). Melphalan-resistant multiple myeloma: results of treatment according to the M-2 protocol.. Cancer Chemother Pharmacol.

